# Application of Distributed Fibre Optical Sensing in Reinforced Concrete Elements Subjected to Monotonic and Cyclic Loading

**DOI:** 10.3390/s22052023

**Published:** 2022-03-04

**Authors:** Yasmin Lemcherreq, Tena Galkovski, Jaime Mata-Falcón, Walter Kaufmann

**Affiliations:** Institute of Structural Engineering (IBK), Swiss Federal Institute of Technology Zurich (ETHZ), Stefano-Franscini-Platz 5, 8093 Zurich, Switzerland; galkovski@ibk.baug.ethz.ch (T.G.); mata-falcon@ibk.baug.ethz.ch (J.M.-F.); kaufmann@ibk.baug.ethz.ch (W.K.)

**Keywords:** distributed fibre optical sensing, strain measurement, reinforced concrete, cyclic loading, initial stress state, bond

## Abstract

Distributed fibre optical sensing (DFOS) is increasingly used in civil engineering research. For reinforced concrete structures, almost continuous information concerning the deformations of embedded reinforcing bars can be obtained. This information enables the validation of basic and conventional assumptions in the design and modelling of reinforced concrete, particularly regarding the interaction of concrete and reinforcing bars. However, this relatively new technology conceals some difficulties, which may lead to erroneous interpretations. This paper (i) discusses the selection of sensing fibres for reinforced concrete instrumentation, accounting for strain gradients and local anomalies caused by stress concentrations due to the reinforcing bar ribs; (ii) describes suitable methods for sensor installation, strain acquisition and post-processing of the data, as well as determining and validating structurally relevant entities; and (iii) presents the results obtained by applying DFOS with these methods in a variety of experiments. The analysed experiments comprise a reinforced concrete tie, a pull-out test under cyclic load, and a flexural member in which the following mechanical relevant quantities are assessed: the initial strain state in reinforcing bars, normal and bond shear stresses, deflections as well as forces. These applications confirm the benefit of DFOS to better understand the bond behaviour, but also demonstrate that its application is intricate and the results may lead to erroneous conclusions unless evaluated meticulously.

## 1. Introduction

The response of structural concrete members is governed by the interaction between the reinforcement and the surrounding concrete. Since the first use of reinforced concrete structures, researchers have been concerned with the understanding and proper modelling of this interaction. However, it was hardly possible to investigate the stress transfer between concrete and reinforcement experimentally due to limitations of the measurement technology available and the fact that the interface remains difficult to access without influencing its structural behaviour. The latest developments in measurement technology, especially in fibre optical sensing, bear the potential to greatly improve the understanding of this interaction.

**Figure 1:**
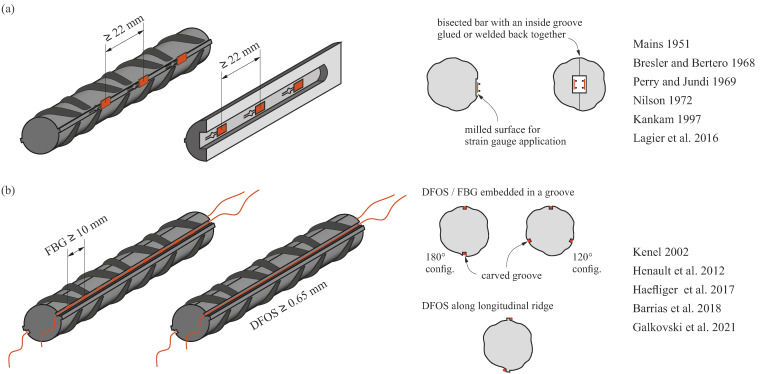
Methods to directly assess the strain distribution of reinforcing bars embedded in concrete (as examples without claiming completeness): (**a**) strain gauges glued on and inside a reinforcing bar [1,2,3,4,5,6]; and (**b**) fibre optical sensors installed on a reinforcing bar using either fibre Bragg gratings (FBG) [10] or Distributed Fibre Optical Sensing (DFOS) [14,18,24,28].

Traditionally, the investigation of the concrete-to-steel interaction was carried out on pull-out tests with small bonded lengths. Under the assumption of nominal bond shear stresses of constant magnitude over the bonded area, these simple tests allow the deriving of bond stress–slip relationships, which can then be used to model the stress transfer between concrete and reinforcement. Despite being widely used, the traditional experimental setup remains controversial, as it does not represent the stress conditions in an actual structure: for instance, compression fields are generated by the bearing plate used in the tests, and the concrete cover exceeds practical values. Hence, many efforts were undertaken to study the steel strain variation directly in structural concrete elements. By using embedded electrical resistance strain gauges, researchers succeeded in experimentally demonstrating the contribution of the concrete between the cracks (e.g., [1,2,3,4,5,6]). In most experiments, strain gauges were glued to machined surfaces on the reinforcing bar (Figure 1a). To not alter the interface with the instrumentation, some researchers bisected reinforcing bars along their axis, glued strain gauges in a longitudinal groove, and subsequently welded or glued the bars back together. With this labour-intensive and invasive instrumentation method, the strain distribution and bond behaviour could be investigated under more realistic conditions and even beyond the yielding of the reinforcement, whereas in standard pull-out tests, bond failure occurs before the yield strength of the reinforcement is reached. However, this method is costly and limited to large diameters since sufficient space is needed to accommodate the strain gauges and their wires.

With the development of fibre optical sensors, the measurement of strain profiles using a series of strain gauges became less common. Initially, optical fibres were also designed as discrete sensors, typically using fibre Bragg gratings (FBG), in which sensors are written into an optical fibre. Temperature or strain changes over the grating length alter the properties of the reflected light, which is used to quantify the strain or temperature difference. Fundamentals on the functionality of FBG can be found in [7,8]. Such sensors, typically around 10 mm long [9], are comparable to a strain gauge chain with the major advantage of bearing a single wire and being smaller in size. Kenel et al. (2002) instrumented the reinforcement of a four-point bending test by gluing FBG sensors into a 1 × 1 mm^2^ groove carved in the bars by planing. Thereby, they obtained the steel strain distribution along the bar at 10.4 mm spacing, reaching the plastic steel range [10,11]. In a recent study, Kaklauskas et al. (2019) compared the two outlined methods (i.e., strain gauges inside the bar and FBG embedded in a groove on the bar) regarding the reliability and accuracy of the strain distribution [12]. In their tests on reinforcing bars embedded in concrete, the strain gauges and FBG were spaced at 30 mm and 20 mm, respectively. The study attested to both methods good accuracy, with FBG proving by far the more practical method. However, anomalies in the FBG measurement were observed at the transition from embedded to bare bar, which according to the authors of the study, might be caused by altered bond conditions and would need further investigations.

Although FBG is applicable over longer sensing distances and requires a significantly smaller intrusion of the host material, it only provides information at the locations of the sensors, i.e., critical locations might be missed unless a large number of gratings at small spacings is employed as by Kenel et al. [10,11]. However, the latter is very expensive, as one single gauge (grating) costs around EUR 100. The development of the Rayleigh backscatter-based coherent Optical Frequency Domain Reflectometry (c-OFDR) overcomes this shortcoming: inexpensive standard glass fibres can be used to obtain continuous strain measurements. The Rayleigh scatter is caused by a random fluctuation in the refraction index of the fibre and is an intrinsic property of each fibre. The collected scatter shows a spectral shift compared to the initial state when the fibre is exposed to an external change in strain or temperature [13,14]. Essentially, the fibre core acts as a sensor in c-OFDR, providing information along the entire fibre length. A high spatial resolution characterises this technology and allows for unmatched strain measurement with gauge lengths down to 0.65 mm. Moreover, c-OFDR enables the simultaneous acquisition of global (average over numerous virtual gauges) and local deformation measurements.

The c-OFDR based strain measurement has recently found broad application in civil engineering research [15], where it is commonly referred to as Distributed Fibre Optical Sensing (DFOS). While DFOS encompasses other distributed sensing methods, such as Optical Time Domain Reflectometry based on Raman-/Brillouin-backscatter, this acronym is used in the following synonymously with c-ODFR. In reinforced concrete structures, the distributed strain measurement of the reinforcement has been used to study the reinforcement concrete interaction with unprecedented detail [16,17], in addition to quantification of other phenomena such as crack [18,19,20,21], shear [22] and load–deformation behaviour [23]. Hereby, prior knowledge of the crack location or any other critical behaviour to instrument the reinforcement accordingly is no longer necessary. To validate DFOS measurements, two approaches were followed: (i) comparison of direct results to local deformations of strain gauges [20,24]; (ii) comparison of integrated DFOS strain values to crack widths or deflections measured with LVDTs or DIC [19,22]. In a further application, shrinkage strains of the uncracked specimens were measured to account for their influence on the response of the reinforced concrete ties [25], and code predictions for shrinkage and creep strains were verified using DFOS [26]. Recently, Monsberger and Lienhart (2021) employed DFOS to extract the curvature and bending moment in structures by double integration of the obtained strains [27].

Despite the versatility of DFOS, most current applications are limited to small deformations and monotonic loading. The limitation to small deformations is provided by the measurement ranges of the spectrometers currently in use (typically up to 12,000 µm/m). Galkovski et al. (2021) reported the possibility to extend the measurement range of the spectrometer by setting intermediate reference states and superposing the data afterwards [28]. Their method enables measurement of strains up to 25,000 µm/m. Note that the latter is particularly relevant when reinforcing bars exhibiting a yield plateau are instrumented, where local strains corresponding to the Lüders strain, typically exceeding the measurement range of 12,000 µm/m occur immediately after reaching the yield limit.

Only a few applications of DFOS under repeated and high cyclic loading are known to the authors. Broth and Hoult (2020) used nylon-coated fibres to assess the deformation of slender and deep reinforced concrete beams subjected to dynamic loads (3600 cycles of three and four-point bending applied at 1 Hz). The DFOS revealed a localised increase in strains with the number of cycles, attributed to the loss of tension stiffening. The dynamic loads and subsequent loading to failure did not cause an impairment in the DFOS performance [29]. Fernandez et al. (2021) assessed the long-term performance of robust fibre optical cables with a steel tube embedded in concrete and subjected to different loading conditions. By comparing integrated values (i.e., deflection and crack width) to DIC measurements, they concluded that the DFOS measurement remained stable over time [30]. The deterioration of bond properties with increasing load cycles was investigated in the first application of DFOS with high cyclic loading [16]. In this study, the redistribution of bond shear stresses over the bond length was observed. However, an increase in anomalous measurements with the load cycles was reported.

The present paper discusses the particularities of using DFOS to instrument reinforcing bars in RC. This comprises practical recommendations for new and experienced users. Procedures are proposed for instrumenting embedded reinforcing bars with DFOS, including methods to avoid damaging the fibres while casting. Furthermore, methods for the determination of steel stresses in the reinforcing bars, nominal bond shear stresses between concrete and reinforcing bars, bond-slip and crack widths are presented, discussing potential problems and limitations. These comprise (i) the interpretation of local phenomena typically unaccounted for by conventional models, but captured by high-resolution DFOS, and the quantification of their impact on the above-mentioned values, and the global response of the elements, (ii) the difficulties of dealing with signal anomalies (i.e., noise and outliers) and establishing transparent post-processing methods to eliminate them, and (iii) accounting for the initial strain states that inevitably exist in structural concrete. Furthermore, the challenges arising in long-term and high cyclic tests are addressed.

## 2. Research Significance

The unprecedented high spatial resolution of DFOS sheds light on local and global phenomena, some of which might not be part of the intended investigations nor anticipated so that this advantage might turn into a drawback if the analyser is no longer able to interpret or trust the obtained strain measurements. The development of measurement anomalies, such as noise and outliers, renders proper interpretation of the results more challenging. Thus, it is not surprising that questions concerning measurement reliability arise with the increasing use of this technology. The present paper discusses the accuracy and determines the challenges and limitations on simple elements in which the deformation response is largely understood and verifiable. Once the challenges regarding application methods and limitations are mastered, DFOS can be incorporated into complex experiments and structures with due care, providing valuable insights into their internal strain distributions and thus an understanding of their mechanical response.

## 3. Selection of Sensing Fibres for Reinforced Concrete Instrumentation

The following section investigates the ability of various single-mode fibres to accurately measure some particular effects observed in reinforcing bars embedded in concrete, such as strain discontinuities at concrete cracks and strain gradients caused by the contribution of concrete in bearing tensile forces between cracks. Furthermore, the performance of these fibres under repeated loading is studied. Lastly, the strain profiles obtained with the fibres on reinforcing bars embedded in concrete will be analysed. These issues are discussed based on experimental results but without claiming generality or completeness.

All the DFOS data presented in this paper were acquired with the optical distributed sensor interrogator ODiSI-6104 supplied by Luna Innovations [31]. The optical fibres were connected to the interrogator via standoff cable and standard length remote module. The spatial resolution (gauge pitch) is customisable. It was set, unless stated otherwise, to the smallest possible value (0.65 mm) following the recommendations of the supplier to obtain the best possible measurement quality. The measurement frequency varied as it depends on the chosen gauge pitch and the length of the fibre optical sensor. Table 1 gives an overview of all the tests discussed in this paper and the fibres employed in each test. A two-component epoxy (Sikadur-52, Young’s Modulus 1.8 GPa [32]) is used for gluing. Although not explicitly studied in this paper, previous unpublished studies of the authors comparing different adhesives have shown its suitability. For further studies on the matter, the reader is referred to [33,34].

### 3.1. Suitability for Measuring Strain Gradients, Discontinuities and Repeated Loading

Much research has been carried out to investigate the suitability of various coating types, e.g., [19,23,25]. Two types of coating are commonly used: (i) polyimide coating, which is chemically bonded to the fibre cladding and has high stiffness, exhibiting minimal slip to the glass fibre; and (ii) acrylate coating, which is mechanically bonded to the fibre cladding and exhibits significantly more slip. This section investigates the ability of fibres with these two different types of coating to reliably measure strain gradients and discontinuities and how repeated loading affects their measurement quality.

#### 3.1.1. Experimental Setup and Specimen Layout

For this purpose, a steel plate with changing cross-sectional geometry was tested under direct tension. The specimen was cut from a 10 mm thick S355 J2 steel plate. Its total length measured 1345 mm and the cross-section width varied between 30 and 120 mm. The cross-sectional area and hence, the resulting strains were chosen such that they represent typical strain conditions in a structural concrete element under tension. Discontinuities of the cross-section were used to simulate cracks, and a tapered cross-section simulated the tension stiffening effect. The advantage of using a steel plate is that the gradients and discontinuities do not change with the load cycles (provided that no fatigue cracks appear), whereas in an embedded reinforcing bar, new cracks may appear, and tension stiffening may also be affected by the deterioration of bond with repeated loading [3,16,35].

**Figure 2:**
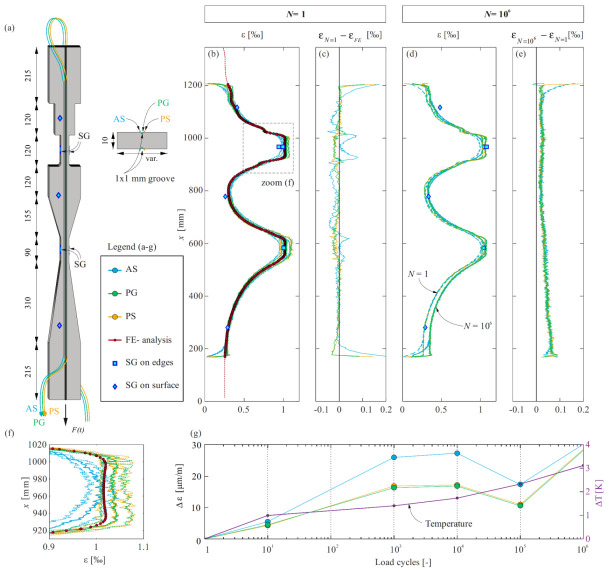
Influence of discontinuities, strain gradients, and repeated load cycles on DFOS measurements in a steel plate: (**a**) specimen layout with instrumentation (AS: acrylate FOS on the surface; PG: polyimide FOS in a groove; PS: polyimide FOS on the surface; SG: strain gauge)—dimensions in [mm]; (**b**) strain profiles and (**c**) difference between the DFOS strains and the strains simulated at the upper load of the first load cycle; (**d**) strain profiles and (**e**) difference between the DFOS strains at the upper load of the first and the millionth load cycle; (**f**) zoom to the marked window in (**b**); and (**g**) strain difference at point *x* = 967 mm and temperature evolution over the applied load cycles.

The instrumented steel plate was tested under repeated tensile loading. Figure 2a displays the layout and instrumentation of the specimen with fibre optical sensors (FOS). Both sides of the specimen contained a longitudinal groove of 1 mm x 1 mm dimensions, in which one polyimide coated fibre was glued (PG) using a two-component epoxy (Sikadur-52 [32]). Next to the groove on both plate sides, another polyimide-coated (PS) and an acrylate coated (AS) fibre were glued on the surface, using the same adhesive with an added filler for better workability. Hence, the influence of various applications (i.e., glued inside a groove or on the surface) was investigated in addition to the influence of strain gradients, strain discontinuities, and fibre coatings. As this test represented a proof of concept, the setup was kept simple. However, a representative number of cycles was applied to gather information on the performance of DFOS under fatigue loading. The results were compared to conventional strain gauges of type TML PFL-10-11 (positioned according to Figure 2a) and linear elastic finite element calculations. The latter were conducted with the software Abaqus/CAE 2019 [36], assuming a Young’s Modulus of 200 GPa. The mesh size was varied according to the geometry of the plate, with a refined mesh at the smallest cross-sections and discontinuities.

The test was carried out in a universal testing machine of the type Schenck 480 kN. The upper and lower load were set to 60.5 kN and 20 kN, respectively. The loading type altered between sequences of monotonic and dynamic loading. The monotonic loading was applied under displacement control with a constant rate (0.01 mm.s^−1^). In contrast, regarding the dynamic cycles, loads of the same intensity were applied in a sinusoidal form at a frequency of 5 Hz under force control to ensure a constant load amplitude. The applied force, the machine displacement and the gauge strains were recorded continuously, at a rate of 100 Hz in the dynamic cycles and 5 Hz in the monotonic cycles. The DFOS measurements (gauge spacing of 0.65 mm and a sampling frequency of 4.2 Hz) were only carried out during the monotonic cycles. A total of one million cycles were executed over seven days. The ambient temperature was recorded throughout the test duration.

#### 3.1.2. Results and Discussion

Figure 2b illustrates the strain profile obtained with the different DFOS configurations, the discrete strain gauges values and the linear elastic calculation at the upper load level (*F* = 60.5 kN). The DFOS data shown here are raw data that were down-sampled to 1 Hz as were the other measurements. The difference between the DFOS strain profiles and the one obtained from the simulation is provided in Figure 2c. The results confirm the observations reported in the companion paper [28]: at the start and end of the length of interest, the polyimide coated fibres measured a sharp strain increase (i.e., short activation length), while the strains measured by the acrylate-coated fibres showed a gradual increase to the same level as the other two fibres (i.e., long activation length). The slip between fibre and coating causes this variance in the activation length and is also the reason why the acrylate coated fibre is unable to reproduce sharp changes in the strain profile (refer to Figure 2f): unlike both polyimide-coated fibre types (PG and PS), the acrylate-coated fibre could not reproduce the plateau in the strain profile and measured lower maximum strains.

Figure 2d shows the strain profile at the upper load at the first and the millionth cycle. The strain profiles were similar, and none of the DFOS configurations displayed anomalous values. Thus, the applied load cycles did not affect the measurement quality. However, a shift in the strain profiles was observed. The difference between these strain profiles $\varepsilon_{N=10^{6}}-\varepsilon_{N=1}$ is shown in Figure 2e. The strain difference was almost uniform in the upper part (*x* > 700 mm) and increased gradually toward the lower end. To investigate the reason behind this small but non-negligible change, the development of the strain difference at around *x* = 967 mm over the load cycles was studied. Figure 2g compares the development of strain differences at this point of all configurations to the ambient temperature change. Both PG and PS displayed a strain difference of approximately 28 μm/m from the first to the millionth cycle, which corresponds to a temperature change of 3.4 K (assuming an expansion coefficient of 8.32 10^−6^ K^−1^ based on the ODiSI-A user guide [37]), whereas AF exhibited a slightly higher value of 3.6 K. These values correspond well with the measured ambient temperature difference of 3.1 K at *N* = 10^6^, as well as with the variation over the cycles. The reason for the gradient in Δε along the specimen might be that the repeated loading caused the piston, which was situated on the bottom end, to warm up and heat was transferred to the steel specimen.

This example illustrates the ability of DFOS to measure strain gradients and discontinuities, which is essential in order to obtain meaningful measurements in structural concrete elements. The polyimide-coated fibres are recommended as they demonstrate a higher accuracy, as reported in [23,28]. The results highlight the necessity of temperature compensation in long-term tests. The compensation for temperature changes in the host material itself is, however, challenging. Concerning repeated loading, the different coatings and application methods did not show a degradation of the measurement quality after one million load cycles. This is in contrast to the findings from [16], where an increase in anomalous measurements with cycles was reported in measurements on reinforcing bars embedded in concrete. These anomalies may thus be related to the interaction of the reinforcing bars and the surrounding concrete.

### 3.2. Effect of Reinforcing Bar Ribs

As outlined in the previous section, polyimide-coated fibres are better suited to measure strain gradients than acrylate-coated fibres and show a higher accuracy. This confirms the findings of Mata-Falcon et al., who also studied the performance of polyimide-coated and acrylate-coated fibre optical sensors by investigating their ability to measure strain gradients on a steel plate with variable cross-sections [23], similar to the experiments presented in Section 3.1, concluding that polyimide-coated fibres are preferable precisely due to their high sensitivity. On the other hand, it has been argued that the high sensitivity of polyimide-coated fibres was unfavourable for the investigation of embedded reinforcing bars [19] since strains exceeding the plausible range were measured, exhibiting jagged distributions with high measurement noise. Hence, it was concluded that nylon-coated fibre optical sensors, measuring smoother distributions and almost free of local peaks, were preferable.

To address these contradicting conclusions, the authors investigated both types of fibre coatings on bare reinforcing bars and on reinforcing bars embedded in concrete. Based on the investigations on bare reinforcing bars, which are presented in a companion paper [28], it was concluded that fibre optical sensors with high sensitivity, i.e., polyimide-coated fibres, can measure strain gradients and local extreme values more reliably, as they have a significantly shorter activation length. Furthermore, it was found that the jagged distributions of strains measured by such fibres are no artefact or measurement anomaly but rather caused by the presence of ribs and can be managed in the post-processing. These findings for bare bars are verified for cast-in reinforcing bars in the following.

#### 3.2.1. Experimental Setup and Specimen Layout

**Figure 3:**
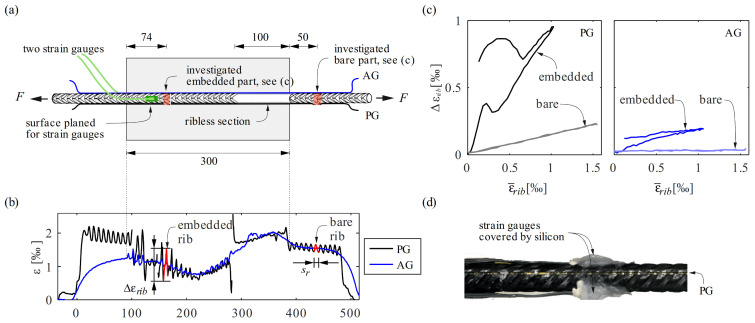
Embedded cold-worked reinforcing steel bar with a ribless section tested under direct tension (specimen d14c): (**a**) geometry and instrumentation (AG: acrylate-coated FOS in a groove, PG: polyimide-coated FOS in a groove, and strain gauges)—dimensions in [mm]; (**b**) DFOS strain distribution at the upper load; (**c**) local strain difference at the ribs marked in (**b**) against the mean strain within the rib lengths for AG and PG; and (**d**) detail of the mounted strain gauges and the fibre optical sensor PG before casting.

A short reinforcing bar embedded in concrete was tested in direct tension to investigate the fibre coating influence. This specimen, designated as d14c, was tested within the scope of a Master’s Project Thesis supervised by the authors [38]. Figure 3a shows the reinforced concrete (RC) specimen with a centrically placed bar made of cold-worked steel. The dimensions of the concrete were *L* × *b* × *h* = 300 × 150 × 150 mm^3^. Self-compacting concrete with a maximum aggregate size of 16 mm was used, with an average compressive strength of 38 MPa after 28 days (Sikacrete ^®^-16 SCC [39]). The steel bar had a nominal diameter of 14 mm. It was instrumented with an acrylate-coated and a polyimide-coated fibre optical sensor (AG and PG), respectively, both glued inside longitudinal grooves in a 180° configuration. The grooves were deliberately carved (by planning) such that they crossed the ribs at their peaks. The length of interest was 500 mm, extending 100 mm outside the concrete at both ends. The bar surface was milled over a length of 20 mm, from *x* = 125 to 145 mm, on opposite sides to mount two strain gauges. The strain gauges, located at a distance of 40 mm from the concrete edge, were sealed with a layer of silicone (Figure 3d). Finally, ribs were removed using a lathe to obtain a cylindrical bar along 100 mm of the embedded part, allowing to study the influence of the ribs on the DFOS data.

The specimen was tested in displacement control at 0.04 mm.s^−1^ loading rate in a universal testing machine of type Schenck 480 kN. A total of 11 loading cycles in the elastic range of the reinforcing bar, with an upper load of *F* = 46 kN ≈ 0.6 *F_y_* were applied, followed by two cycles in the plastic range before the specimen was loaded to failure. Only data from the first elastic cycle are presented and discussed here.

#### 3.2.2. Results and Discussion

Figure 3b displays the strain distribution of the polyimide-coated (PG, black) and acrylate-coated (AG, blue) fibre optical sensors, respectively, at the upper load of the first cycle. It reveals bending action on the left side caused by the clamping and geometry tolerances of the bar. Mean strains and local peaks at the ribs measured using the PG sensor were slightly higher in the bare bar on the left side than on the right side. Over the embedded length, local peaks were more pronounced in the vicinity of the left concrete edge and correlated with the increase of the relative slip between concrete and reinforcement. The local peaks seemed to increase with increasing slip. The ribless part biased the measurements in the right part of the embedded length and the latter effect was thus less pronounced on that side. In the measurements of AG, no significant local peaks could be observed in the bare parts, and only slight oscillations appeared in the embedded length. None of the fibres measured significant strain peaks in the ribless part. The sections where the strain gauges were located showed much smaller local peaks at the ribs. Moreover, the strain distribution had a local plateau, indicating that no load was transferred there to the concrete. The same applied to the embedded region without ribs. The much higher activation length of the acrylate-coated fibre was evident also in this test, particularly in the bare parts.

The effect of the local disturbance by a rib is investigated in Figure 3c for the sections marked red in Figure 3a. These sections each span one rib spacing $s_{r}$, one of them being located in the embedded part and the other on the right part outside the concrete. Figure 3c presents the strain difference $\Delta \varepsilon_{rib}$ (difference between maximum and minimum strain measured within the length $s_{r}$) against the mean strain over the length $s_{r}$ in these sections for both sensors PG and AG for the first loading–unloading cycle. Both sensors displayed a smaller strain difference in the bare part compared to their embedded region. The sensor AG did not register a relevant strain difference in the bare part at all. In the embedded region, an increase in $\Delta \varepsilon_{rib}$ with increasing mean strains could be observed for both sensors. However, the strain differences $\Delta \varepsilon_{rib}$ measured by the sensor PG were an order of magnitude higher than those obtained from the sensor AG. In contrast to the bare region, the peaks in the embedded region exhibited a hysteresis between loading and unloading.

The wavelength of the undulations coincides with the rib spacing, and local peaks occur systematically at the inter-rib areas, as clearly demonstrated by the polyimide-coated fibre in this test, and thus cannot be considered as random noise. On the other hand, as these peaks also appear in the bare parts with ribs—though with a smaller amplitude–, attributing them to bond action alone [17] may be misleading. Specifically, assuming that the height of the peaks can be translated directly into a locally acting force between a single rib and the surrounding concrete is questionable: the local peaks appear to increase with increasing slip, but not necessarily only with increasing load. To conclusively understand this behaviour, including the effects of bond, the reinforcing bar could be modelled as a three-dimensional body, which is; however, beyond the scope of this paper.

This test demonstrates that the use of conventional strain gauges to measure reinforcing steel strains inside concrete produces significant disturbances in the deformation behaviour of the reinforcement as, locally, bond is lost over the instrumented length. This is caused by the milled surfaces to accommodate strain gauges and the silicon employed to seal them (Figure 3d).

Furthermore, the suitability of polyimide-coated fibre optical sensors for applications in reinforced concrete members is demonstrated by the test. The mechanical origin of the jagged strain distribution can be attributed to the presence of ribs. Therefore, measurements with polyimide-coated fibres can be processed with a suitable filter to obtain mean strains over a rib spacing, which are representative for structural concrete applications while maintaining a short activation length and a high sensitivity [28]. Using fibre optical sensors with a slipping coating, such as an acrylate coating, thus cannot be justified by the advantage of obtaining smooth results.

## 4. Application of DFOS on Embedded Reinforcing Bars

Specific procedures for the instrumentation of bare reinforcing bars, data acquisition, some general aspects for validations, and the determination of steel stresses are published in the companion paper [28]. These are equally valid for measurements in structural concrete elements. This section outlines the most important aspects to be considered when embedding DFOS-instrumented reinforcing bars in concrete, as well as methods for determining bond shear stresses, acting forces, bond-slip and crack widths.

### 4.1. Installation of Sensors

For the measurement of strains in embedded reinforcing bars, it is recommended to use polyimide-coated single-mode fibres with a thin coating. Their small size produces a negligible alteration of the interface between concrete and steel. The fibres can either be placed in a groove or attached along the longitudinal ridge. The authors prefer the first configuration as (i) the fragile fibre is protected from damage during casting and compaction, and (ii) it was observed in some cases, when glued directly on the bar, the adhesive cleaved to the concrete and delaminated from the bar. In other studies, a Teflon layer [17] or a silicon coating [19,33] was applied on the adhesive to prevent contact with the concrete. However, according to the authors’ experience, when embedded in a groove with sufficient depth and filled with a suitable adhesive, no further protection is needed.

**Figure 4:**
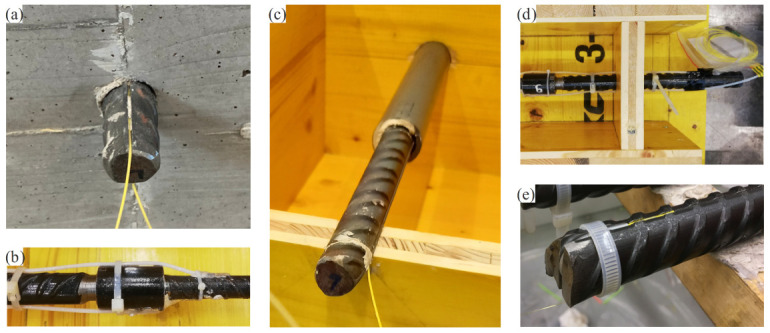
Examples of instrumenting embedded reinforcing bars with DFOS: (**a**) leading out the fibre with simple protection; (**b**) passing couplers; (**c**) placing an instrumented reinforcing bar in the formwork; (**d**) leading out the fibres with additional protection and (**e**) instrumentation of bar ends (anchorage).

The bars should be instrumented with at least two fibres arranged on opposite sides to capture potential bending effects. If the specimen and reinforcement layout permits, it is recommended to lead the fibre ends out of the concrete at a suitable location, i.e., away from areas with high stress concentration, such as supports or load introduction regions. The parts of the fibre located outside the formwork during casting should be protected from mechanical damage (e.g., by a protective tube over the bare fibre and wrapping it in plastic bags). Figure 4a–e display some examples of instrumented embedded bars (b–e before casting). Taking the fibre ends out of the concrete renders it easier to check their integrity visually (e.g., with a red testing laser) and, in the event of a break, to remobilise lost measuring sections by splicing a new connector to them. However, the reference state of the bare reinforcing bar measured before casting is no longer valid in such cases, as the fibre optical sensor needs to be reinstalled in the software. If the termination of the fibre is achieved with coreless glass fibre, it can also be placed inside the concrete. However, this requires appropriate protective measures and reduces the measuring system redundancy.

In anchorage investigations, it would affect the structural behaviour and the measurement quality if the fibre was led out of the concrete at the embedded bar end, where large relative displacements between the reinforcing bar and the surrounding concrete occur. In such cases, it is preferable to loop the fibre around the bar end in a protective plastic tube that runs inside an indentation connecting the grooves on either side of the reinforcing bar at its end (Figure 4e).

### 4.2. Strain Acquisition and Post-Processing of the Data

Once the concrete is cast, parts of the fibre are no longer accessible. The user should register the coordinates of all relevant points that lay inside concrete beforehand (e.g., the lengths of interest and their relative position to each other; if additional strain gauges or other sensors are present, their position with respect to the fibre; or special points of interest or transitions). The local coordinates within the bar are important, in addition to the position of each bar in the specimen, i.e., in the global coordinate system. Precise positioning and documentation are essential for reproducibility and reliable data post-processing.

Furthermore, from the moment the concrete is poured into the formwork, the reinforcement deforms due to the concrete hardening process (hydration heat) and shrinkage. A reference measurement needs to be set before casting to quantify the resulting initial strain state of the composite element. Such shrinkage strain measurements with DFOS are presented and discussed in Section 5.1.

The fibre optical sensors on cast-in bars usually display more anomalous readings and require more post-processing than on bare steel. The user will usually have to identify and cancel out outliers and mitigate peaks resulting from local phenomena. Based on the analysis in Section 3.2 and the companion work [28], a moving average filter with a window size equal to the rib spacing of the instrumented bar, or a multiple of it, is recommended to smooth the local strain peaks due to the ribs of the reinforcing bars. Other post-processing techniques can be found in the literature, e.g., [40].

### 4.3. Derivation of Slip, Crack Widths, Normal and Bond Shear Stresses

#### 4.3.1. Slip and Crack Width

**Figure 5:**
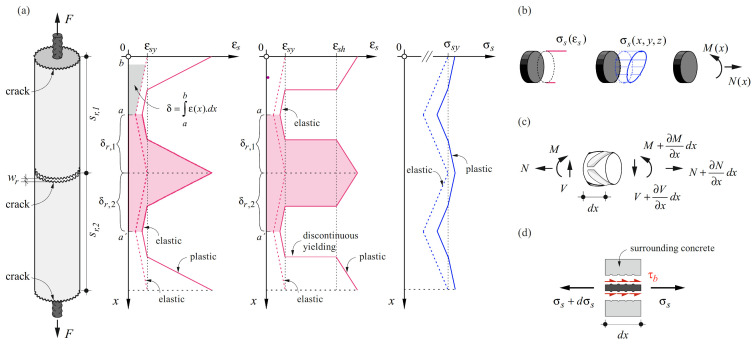
Structurally relevant results obtained from DFOS strain measurements on an embedded reinforcing steel bar: (**a**) simplified steel strain distribution in an RC tie for CW and QST steel, respectively, and the corresponding stress distribution; (**b**) from local stresses to a stress distribution and acting forces on a cross-section; (**c**) equilibrium on a differential steel element; and (**d**) local bond shear stresses based on equilibrium on a differential steel element.

An interesting value that can be obtained directly from the quasi-continuous fibre measurements is the relative displacement between concrete and steel, denoted as slip $\delta \left(x\right)$ (Figure 5a). By integration of the strains starting from a location of zero slip (a, a′), and subtracting the deformation of the concrete, the slip at each point is obtained:

(1) $$\delta \left(x\right)=\int_{a}^{x}\varepsilon_{s}\left(x\right)\cdot dx-\int_{a}^{x}\varepsilon_{c}\left(x\right)\cdot dx$$

In a reinforcing bar embedded in concrete and monotonically loaded in tension, the slip vanishes at the locations of the global strain minimum between two adjacent cracks. Note that due to its low tensile strength, the deformations of the uncracked concrete are very small compared to those of the steel and can usually be neglected.

Adding up the slip from the adjacent crack elements on either side of a crack (obtained by integrating over $s_{r,1}/2$ and $s_{r,2}/2$, respectively, as depicted in Figure 5a) leads to the crack width:

(2) $$w_{r}=\delta_{r,1}+\delta_{r,2}$$

Slip and crack width, as integral values, are less prone to variations resulting from different post-processing and smoothing methods than steel stresses and particularly bond shear stresses, as outlined in the following sub-section. However, defining the location of zero relative slip is challenging during unloading, as is the calculation of (residual) crack openings in such cases: with the decrease of the applied load, slip reversal occurs starting from the crack, along with a potential deterioration of the bond shear stresses [41,42]. These effects lead to an irregular residual strain profile after complete unloading, making it difficult to determine slip and crack width reliably.

#### 4.3.2. Normal Stresses of Reinforcing Steel

Once the acquired strain data have been post-processed, it can be employed to determine the stresses in the reinforcing bars, provided that the stress–strain characteristics of the latter are known. Commonly, such constitutive laws for reinforcing bars are defined using the elongation of a base length containing a multitude of ribs. DFOS, on the other hand, distinguishes between strains at and between the ribs, but as observed by Galkovski et al. [28], mean local strains averaged over a length corresponding to the rib spacing coincide with the global strains. Hence, the virtual gauge spacing needs to be averaged over one rib spacing or a multiple of it to determine stresses from strains obtained by DFOS using common constitutive laws.

**Figure 6:**
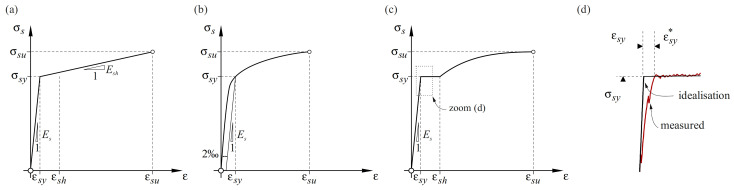
Stress–strain characteristics of reinforcing steel in uniaxial tension: (**a**) bilinear idealisation; (**b**) idealisation for cold-worked (CW) steel; (**c**) idealisation for quenched, self-tempered (QST) steel and (**d**) comparison of the idealisation for QST to author’s test data—the visualisation is limited to the marked area in (**c**).

For a realistic translation of strains into stresses, accurate knowledge of the stress–strain characteristics of the instrumented reinforcing bar is essential. Overly simplified constitutive models lead to biased results. Figure 6 illustrates some commonly used stress–strain relationships for reinforcement with varying degrees of accuracy. The first model (Figure 6a), a bilinear idealisation, presents a strong simplification that has its justification in engineering practice but is inappropriate when accurate stress calculation is essential and in any test where strains exceeding the yield limit are expected. Combining the high-resolution DFOS technology with such a crude idealisation cannot be justified.

If the constitutive law of the reinforcing bar has been determined by material testing, the stresses can essentially be obtained from the strains directly using the experimentally observed stress–strain relationship, suitably smoothed where appropriate and fitted to the average behaviour if several material tests have been carried out. However, this requires knowledge of the experimental stress–strain curve of each reinforcing bar and cannot be used for comparison with model predictions, which are typically based on few parameters, i.e., Young’s modulus $E_{s}$, yield strain $\varepsilon_{sy}$, yield strength $\sigma_{sy}$, and strain at the onset of hardening $\varepsilon_{sh}$ if a yield plateau is present.

Alternatively, the model proposed by Ramberg and Osgood [43] (Figure 6b) can be employed for cold-worked (CW) steel, which lacks a yield plateau:

(3) $$\varepsilon_{s}=\frac{\sigma_{s}}{E_{s}}+{\left(\frac{\sigma_{s}}{k_{c}}\right)}^{\alpha }\;\mathrm{with}\;\alpha =\frac{\ln[(\varepsilon_{su}-\sigma_{su}/E_{s})/k_{a}]}{\ln(\sigma_{su}/\sigma_{sy})}\;\mathrm{and}\;k_{c}=\frac{\sigma_{sy}}{k_{b}^{1/\alpha }}$$

The parameters $k_{a}$ and $k_{b}$ are equal and correspond to the strain at the yielding point; they can, e.g., be fitted to experimental data by the least-squares method, which typically provides good agreement with the experimental curves over the entire strain hardening range. Since Equation (3) cannot be solved for $\sigma_{S}$ analytically, the stresses have to be determined numerically. Other models, such as proposed by Menegotto-Pinto [44], may be used as well.

The deformation behaviour of steel reinforcing bars exhibiting a yield plateau, nowadays typically quenched and self-tempered (QST) straight bars, is accurately reproduced by the following expressions [45]:

(4) $$\sigma_{s}=\varepsilon_{s}\cdot E_{s}\;\;\;\;\;\;\;\;\;\;\;\;\mathrm{for}\;0<\varepsilon_{s}\leq \frac{\sigma_{sy}}{E_{s}}$$

(5) $$\sigma_{s}=\sigma_{sy}\;\;\;\;\;\;\;\;\;\;\;\;\;\mathrm{for}\;\frac{\sigma_{sy}}{E_{s}}<\varepsilon_{s}\leq \varepsilon_{sh}$$

(6) $$\begin{array}{c} \sigma_{s}=\sigma_{sy}+\left(\sigma_{su}-\sigma_{sy}\right)k_{c}\left(1-e^{(\varepsilon_{sh}-\varepsilon_{s})/\alpha }\right)\\ \mathrm{where}\;\alpha =k_{a}\left(\varepsilon_{sh}-\varepsilon_{su}\right)/(\varepsilon_{sh}-k_{b})\;\;\;\;\;\;\;\;\mathrm{for}\;\varepsilon_{sh}<\varepsilon_{s}\leq \varepsilon_{su} \end{array}$$

The parameters $k_{a},\;k_{b}$ and $k_{c}$ can be fitted to the experimental data. The corresponding stress–strain relationship is displayed in Figure 6c. Another suitable model can be found in [46]. The yield plateau and the gradual decrease of the hardening branch slope with increasing strain are captured well. Still, experimentally determined curves will deviate more or less from this idealisation. In particular, even for reinforcing bars of the same steel grade, the length of the yield plateau may vary significantly, and the transition from the linear elastic to the ideally plastic part in the model, i.e., at the onset of yielding, does not capture a flattening of the stress–strain response frequently observed experimentally in QST reinforcing bars. Specifically, the actual strain $\varepsilon_{sy}^{*}$ at the onset of yielding (defined here as the point where the strains start increasing with almost no change in stress) is significantly higher than the theoretical value of the yield strain $\varepsilon_{sy}$ (defined as the ratio of the dynamic yield strength, calculated considering the nominal bar diameter, to the secant modulus) (see Figure 6d). This deviation can be explained by the interaction of distinct material layers with different microstructures present in QST reinforcing bars: when the perlitic/ferritic core, which has a pronounced yield plateau, reaches its yield limit, the outer martensitic layer, without yield plateau but having a higher yield limit, is still elastic. This causes the slope of the stress–strain curve to decrease before a clear yield plateau is reached [47].

The parameters defining the material models should be determined in material characterisation tests, ideally on bars prepared identically as those used in the actual experiments (i.e., with grooves to accommodate optical fibre sensors). Still, the material parameters are subject to scatter and additionally depend on the methods used in the material characterisation. For instance, the yield limit and the tensile strength depend on the applied loading (displacement) rate, with relevant variations even within the strain rates allowed in standards for uniaxial tension tests on reinforcing bars [48]. Depending on the loading rates expected in the actual experiments, either the dynamic or the static yield limit and tensile strength may need to be employed to define the constitutive law. A method to account for strain rate dependency is proposed in [48]. While these effects basically also need to be accounted for when using traditional discrete strain gauges, they become more relevant when using DFOS due to the higher resolution and the possibility to obtain derivatives of the steel stresses with respect to the bar axis, which is sensitive to the shape of the stress–strain relationship.

Once the steel stresses have been obtained from the strain data, they can be used to determine the forces acting in instrumented parts of the tested specimens. To this end, the geometry and the loading type must be known. For instance, in elements (such as reinforcing bars) subjected to normal forces and uniaxial bending, which are instrumented with at least two fibres, following the common hypothesis of Euler–Bernoulli, a plane can be fitted to the measured strains and subsequently, the stresses in the entire cross-section can be determined based on the procedures outlined above. Integration of the stresses then provides the normal force and bending moment acting on the cross-section (see Figure 5b). Finally, force equilibrium on an infinitesimal bar element (see Figure 5c) provides the shear force:

(7) $$V=\frac{dM}{dx}$$

This allows, e.g., determining the contribution of reinforcing bars to shear force transfer in beams, also known as dowel action [17]. Furthermore, knowledge of the strain plane of the bar is sufficient to assess the curvature of the bar for the structural member and loading under investigation; note that in general cases with biaxial bending, at least three fibres are required to determine the strain plane.

#### 4.3.3. Bond Shear Stresses

The load transferred from reinforcing bars to the concrete and vice versa is typically modelled by means of nominal bond shear stresses $\tau_{b}$, which are assumed to be uniformly distributed along the perimeter of the nominal cross-section of the reinforcing bar with diameter Ø (but generally varying along the bar axis). Formulating equilibrium on a differential bar element of length d*x* (refer to Figure 5d), the bond shear stresses amount to:

(8) $$\tau_{b}=\frac{\emptyset }{4}\frac{d\sigma_{s}}{dx}$$

where ${d\sigma }_{s}$ is the differential increase of steel stresses in the bar sections spaced at dx. Hence, bond shear stresses are directly proportional to the variation of normal steel stresses along the bar. Substituting dx by the DFOS sensor spacing and d$\sigma_{s}$ by the difference in the steel stresses at consecutive sensors, bond shear stresses can thus readily be obtained from the variation of steel stresses along the reinforcing bar determined by DFOS.

Three main aspects need to be considered when determining bond shear stresses using DFOS data. First, the local fluctuations in the stress distribution caused by the presence of ribs need to be eliminated, as stated previously. Deriving the stress distribution over the length amplifies the fluctuation and bond shear stresses with meaningless values and even changing signs within one rib spacing may result. Second, however, excessive smoothing of the data must be avoided, as this would attenuate global maxima and minima of the steel stresses, thereby reducing their difference and hence, the magnitude of the bond shear stresses, even if the latter are averaged over a certain length to obtain mean bond shear stresses. Lastly, the conversion of the measured strains to stresses needs to be carried out carefully and account for the particularities of the type of reinforcement used. Figure 5a shows the expected strain distribution along a reinforcing bar consisting of CW and QST steel, respectively, embedded in cracked concrete and the corresponding stress distributions. While the stress distributions are similar (or even coincide if equal bond shear stresses are assumed in both cases), the strain distributions differ strongly at the transition from the elastic to the plastic range. Regarding CW steel, two distinct ranges are identifiable: the elastic range and the plastic range with a decreased stiffness. In contrast, for a QST steel (exhibiting a yield plateau), the strains in the section where the yield limit is reached are expected to increase drastically from the yield limit to the hardening strain [45,49]. This jump, also called discontinuous yielding, can indeed be observed in bare reinforcing bars [28]. However, in embedded QST reinforcing bars, rather than a jump, a transition zone of a certain length, in which the strains gradually increase from the yield strain $\varepsilon_{sy}$ to the hardening strain $\varepsilon_{sh}$, is observed using DFOS measurements. Applying the constitutive law of bare QST reinforcing bars in these transition zones would result in constant stresses $\sigma_{sy}$ and hence, zero bond shear stresses, contradicting the model predictions as observed in Figure 5a. This is further shown and discussed in Section 5.2.

### 4.4. Validation and Plausibility Checks

As outlined above, the post-processed strain data can be used to determine a number of further, structurally relevant results. In addition to yielding insight into the mechanical behaviour—representing the primary goal of determining these values—they are useful to verify the plausibility and accuracy of the DFOS measurements. Some possibilities are listed in the following.

For instance, the measured strains can be compared to discrete strain measurements such as electrical resistance strain gauges, digital image correlation (DIC) based strain data or average strains determined using linear variable displacement transformers (LVDT). In the latter case, mean DFOS strains over the same section as covered by the LVDT must be compared.

By integrating the quasi-continuous strains, deformations can be calculated (similar to the determination of bond-slip) and, if at least two fibre optical sensors are installed, inclinations or curvatures can also be obtained. These values again can be compared to measurements obtained using other instrumentation, such as LVDTs, inclinometers, DIC, or actuator strokes of testing machines.

Finally, the steel stresses obtained from the measured strains can be compared with the stresses determined from applied loads and geometrical information, particularly in cracked cross-sections of reinforced concrete specimens. Precise knowledge of the materials’ constitutive laws is a prerequisite for reliable comparisons.

## 5. Examples of Application in Structural Concrete Experiments

The following section presents examples of the application of DFOS in experiments on structural concrete elements. In the first example, the initial strain state in an embedded reinforcing bar (ERH) and its implications for DFOS measurements are discussed. Subsequently, the derivation of normal and bond shear stresses as outlined in Section 4.3 is applied to a similar specimen (ERV). The third example is a pull-out test subjected to high-cyclic loading, in which DFOS was employed to assess the internal strain and stress state, particularly regarding the load transfer between reinforcing bar and concrete. Those tests are part of a larger experimental campaign conducted to assess the degradation of bond properties within the framework of the first author’s doctoral thesis, whose findings will be published at a later date. For the sake of better readability, the specimens are referred to as ERH, ERV and PO in this paper, rather than S3-ZG-H03, S3-ZG-L03 and S2-PO-06, respectively, as used in the original work. The final example is a beam (Nn) subjected to four-point bending, in which DFOS was applied to assess the curvature, the acting forces and the dowel action in the reinforcing bars. While a discussion of the structural behaviour in these experiments remains beyond the scope of this paper, they are included to show successful applications of DFOS measurement and validate the presented methods.

### 5.1. Initial Strain and Stress State of Reinforced Concrete Elements

#### 5.1.1. Shrinkage-Induced Strains

Shrinkage designates the decrease in the volume of stress-free concrete. It consists of several contributions, with autogenous shrinkage and drying shrinkage being predominant [50]. The former is the result of the chemical hydration of cement without exchange with the environment and mainly occurs in the early stage of hardening. Its value depends on the water–cement ratio and composition of the concrete. The latter, which is dominant in normal strength concrete, is caused by the loss of internal water of the hardened concrete to the environment and thus starts with the formwork removal or the end of curing. Its magnitude depends on humidity and temperature conditions as well as on the element geometry, the exposed surfaces and porosity. The total free (unrestrained) shrinkage $\varepsilon_{cs}$, whose rate decreases over time, can reach values between 0.1 and 0.7‰ [51].

**Figure 7:**
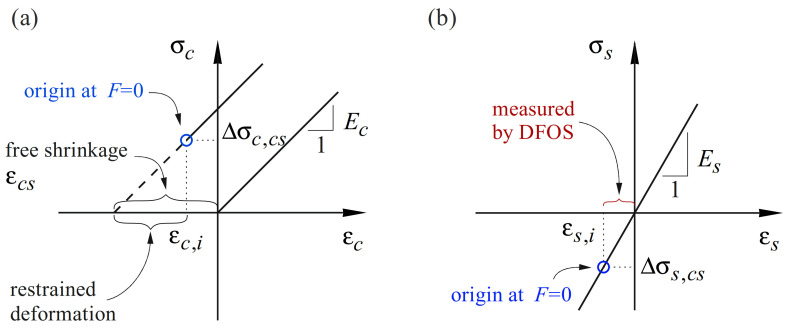
Schematic representation of shrinkage-induced strain and stress in (**a**) concrete and (**b**) reinforcing bar.

In an unreinforced, perfectly unrestrained concrete specimen, shrinkage deformations would not cause any stresses. In reinforced concrete specimens, however, the deformations are internally restrained by the reinforcement even in the absence of external restraint. This internal restraintcauses tensile stresses in the concrete $\Delta \sigma_{c,cs}=E_{c}\cdot \varepsilon_{c,i}$ and, by equilibrium, compressive stresses in the reinforcement $\Delta \sigma_{s,cs}=-E_{s}\cdot \varepsilon_{s,i}$ (Figure 7a and Figure 7b, respectively). In uncracked specimens without external restraint, the initial stress state caused by shrinkage can be determined by equilibrium, assuming that steel and concrete strains coincide $\varepsilon_{c,i}=\varepsilon_{s,i}$. Note that this relationship only applies as long as the tensile stresses in the concrete do not exceed its tensile strength; otherwise, shrinkage cracks occur already in the unloaded state. However, internal restraint by the reinforcement alone is rarely sufficient to cause cracking.

When studying the global response of structural concrete elements, this self-equilibrated initial stress state is often neglected, and the response of the element is assumed to start with the loading. However, the shrinkage-induced stresses can have a pronounced influence on the crack formation and stiffness: the initial tensile stresses in the concrete cause cracking at reduced loads than expected in initially stress-free elements. The corresponding apparent tensile strength must not be confused with the effective concrete tensile strength. Moreover, neglecting the initial strains and stresses caused by shrinkage leads to an underestimation of the concrete tensile contribution between the cracks [52] and an overestimation of the tensile stresses in the reinforcement.

Taking shrinkage stresses and strains into account is challenging, and without measurements on a specimen, only rough estimates are possible. Even if strain measurements are available, determining the initial stresses is not straightforward owing to the complex nature of the time-dependent processes and their dependence on the actual exposure and dimensions of a specimen. Standards provide empirical formulas to approximate free shrinkage, which though is subject to significant scatter. A better approximation is obtained by shrinkage measurements on plain concrete specimens, which are cast using the same concrete and stored in the same conditions as the specimens under investigation.

#### 5.1.2. DFOS Adjustment for Shrinkage-Induced Strains

For structural concrete elements instrumented with DFOS, the initial strains can easily be obtained. Sensors installed prior to casting indicate strain and temperature changes with respect to this reference state (i.e., strain change from bare to embedded state). In contrast to other strain measuring methods, no additional work steps are required.

**Figure 8:**
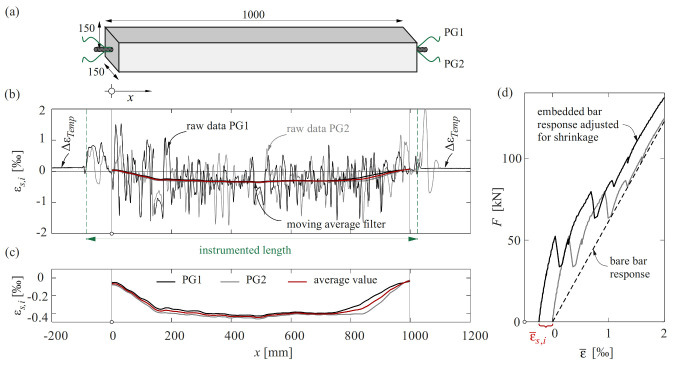
Initial strain state in an embedded reinforcing bar (Specimen ERH): (**a**) geometry and instrumentation (PG: polyimide FOS in a groove)—dimensions in [mm]; (**b**) shrinkage strain measured by DFOS at an age of 150 d (raw data); (**c**) shrinkage strain after post-processing and (**d**) global response of an embedded reinforcing bar subjected to deformation controlled uniaxial tensile loading with and without adjustment for shrinkage.

The measurement of shrinkage strains with DFOS is shown on Specimen ERH, consisting of a single bar with a nominal diameter of 20 mm cast in the centre of a concrete tie of 150 × 150 mm^2^ cross-section and 1000 mm length (Figure 8). The reinforcing bar was instrumented with two polyimide-coated glass fibres (PG) glued inside 1 × 1 mm^2^ grooves in a 180° configuration. The concrete had a maximum aggregate size of 16 mm and a compressive cylinder strength of 29.2 MPa after 28 days. After casting, the specimen was covered with a plastic sheet for curing, and the formwork was removed after seven days. The specimen was then stored under laboratory conditions until testing.

Figure 8b shows the shrinkage strains $\varepsilon_{s,i}$ captured with DFOS at an age of 150 d. The raw data—referring to the initial measurement before casting—exhibited considerable variation. However, a simple moving average filter with a large window size of 130 mm cancels out the fluctuations (Figure 8c). The correlation between the two instrumented sides (e.g., around *x* = 190, 500 and 900 mm) suggests a physical cause behind the observed strain fluctuations. The elapsed time between the reference state (before casting) and the shrinkage measurement can be excluded as a reason since the loose sensor sections outside the concrete did not reveal any anomalous readings. The most plausible explanations are (i) local strain variations between the shrinking cement paste and the aggregates that do not shrink and (ii) internal micro-cracking caused by the resulting restraint stresses. Indeed, non-shrinking particles, such as aggregates and unhydrated cement, restrain the shrinkage of the cement paste, which explains why shrinkage strains of concrete are much smaller than those of cement paste and decrease with the relative aggregate volume (see e.g., [53]). As the gauge pitch used was much smaller than the aggregate size, strain differences between aggregates and cement paste, as well as internal micro-cracks, were captured by DFOS. Further investigations would be necessary to clarify the causes.

In the example given, a change in the ambient temperature was also measured by DFOS. This is recognisable on the vertical shift $\Delta \varepsilon_{Temp}$ in the loose sensor sections outside the concrete (Figure 8b), and was compensated by shifting the data by this amount. This implies the assumption that inside the concrete, temperature changes corresponded to ambient temperature changes. A more accurate approach would be to install a loose fibre (for instance, in a tube) in the concrete, which would separately capture internal temperature developments.

Figure 8c illustrates the shrinkage strains following post-processing and the adjustment for temperature variation (the two lengths of interest and their mean value are only plotted inside the concrete). The smoothed strain profile is in agreement with findings from the literature [26]: a transition length is observed at both ends of the specimen, with shrinkage strains increasing toward the middle. This indicates the development of bond shear stresses in those parts. In between, the strains are almost uniform, and the smoothed steel strains can be used to obtain steel stresses (following the procedure outlined in Section 4.3.2) as well as concrete stresses, assuming strain compatibility in the uncracked concrete, $\varepsilon_{c,i}=\varepsilon_{s,i}$. Note that the latter are caused by the difference between free shrinkage strain and observed strain, i.e., $\sigma_{c,i}=E_{c}\left(\varepsilon_{cs}-\varepsilon_{c,i}\right)$, where $E_{c}$ represents the Young’s modulus of concrete.

For subsequent measurements, it is recommended to set a new reference state at the beginning of testing and superpose the smoothed shrinkage strains in the post-processing. Figure 8d shows such correction of the global response of ERH subjected to uniaxial loading based on DFOS measurements. The applied load is plotted against the average strains $\bar{\varepsilon }$ over the concrete length with and without shrinkage adjustment. The latter is performed by adding the smoothed shrinkage strain to the load-induced strains of each sensor and averaging the obtained strain profile over both sides of the reinforcing bar and the concrete length. It can be observed that neglecting the initial strains would lead to underestimating tension stiffening (the difference between the response of the embedded and the bare reinforcement).

### 5.2. Normal and Bond Shear Stress in Reinforced Concrete Elements

The derivation of normal and bond shear stresses as outlined in Section 4.3.2 is applied to an RC tie (ERV) subjected to uniaxial tensile loading. The specimen had identical dimensions, material properties and instrumentation as ERH (cf. Section 5.1). The reinforcing bar was a straight QST bar of the class B500B according to the Swiss Standard SIA 262:2013. Material characterisation tests were performed on seven bars obtained from the same batch. The material tests were conducted deformation controlled at 0.04 mm.s^−1^ and increased ten-fold after the onset of yielding. The obtained properties are provided in Table 2. The concrete properties were determined on cylinders and cubes after 28 days: compressive cylinder strength $f_{c}=29.2\;\mathrm{MPa}$, tensile strength $f_{ct}=2.7\;\mathrm{MPa}$ (determined with a double-punch test), and Young’s Modulus $E_{c}=28.5\;\mathrm{GPa}$.

In addition to the DFOS, one concrete surface of each RC tie was instrumented with a 3D-DIC system to track the global deformation of the specimen. A speckle pattern consisting of black circular points with a diameter of 1.2 mm was applied using a speckle roller. The images were captured at 1 Hz with two cameras (FLIR 12.3 MP) using 28 mm focal length Zeiss lenses with a baseline of 942.6 mm and a resulting resolution of 3.34 px/mm. The correlation was performed with the software VIC-3D (Correlated Solutions Inc., Colombia, SC, USA, [54]) using a subset size of 21 pixels and step size of 6 pixels. The results were then used to determine the crack patterns and crack widths using the Automated Crack Detection and Measurement (ACDM) software [55].

The specimen ERV was tested at a concrete age of 191 d. The initial stress state (i.e., shrinkage induced stress) was measured before clamping the specimen in the universal testing machine. The load was applied to the reinforcing bar ends deformation controlled at a loading rate of 0.01 mm.s^−1^. During the experiment, the DFOS measurements were tracked continuously. Once the measurement became unstable (i.e., rise in unrealistic readings due to local strains in the order of the measurement range of the spectrometer, see Section 1), the loading was set on hold and a new reference state was measured after two minutes break for steel relaxation as proposed in [28]. This procedure enabled strain measurements beyond the yield limit. The test ended when the DFOS correlation was lost entirely, and the setting of a new reference state was no longer possible.

**Figure 9:**
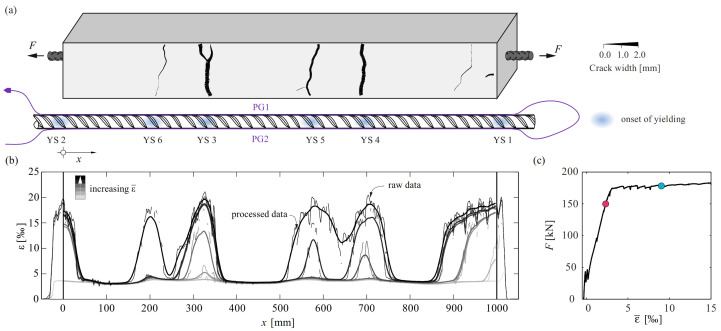
RC tie subjected to uniaxial loading (specimen ERV): (**a**) schematic representation of the tested specimen with the crack patterns and crack widths at *F* = 175 kN (top) measured with DIC, and location of the yield sections (YS) along the bar (bottom); (**b**) DFOS strain distribution along the specimen and (**c**) load–deformation curve.

Figure 9a displays the specimen with the crack widths computed with ACDM at *F* = 175 kN. The locations where yielding started are indicated on the bar also shown in Figure 9a (bottom). Figure 9b exhibits the strain distribution of one sensor along the reinforcing bar for successive increasing load steps starting with the nucleation of the first yield section (YS). The measurements were adjusted for shrinkage as described in Section 5.1. Both raw and smoothed data are given. The latter was obtained by removing outliers, filling the missing values by linear interpolation and applying a moving average over 31 data points, i.e., 19.5 mm. The applied force is plotted against the average steel strains in Figure 9c. The latter was obtained by averaging the strains of both sensors over the concrete length.

**Figure 10:**
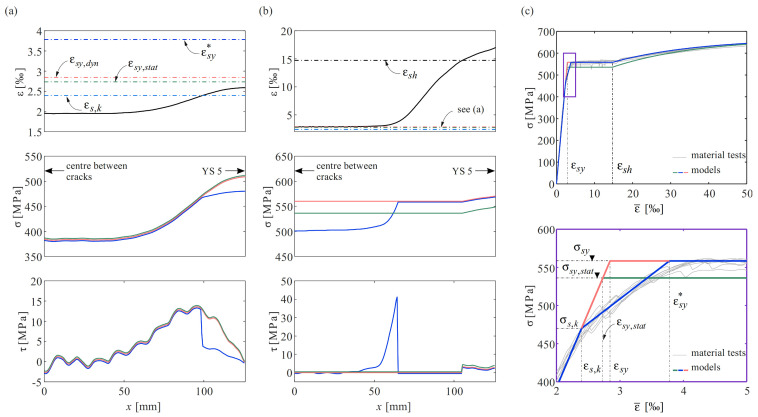
Derivation of normal steel and bond shear stresses from DFOS strain distribution in a half crack element using various idealisation for QST steel behaviour: (**a**,**b**) strains, normal and bond shear stress distribution at *F* = 150 and 178 kN, respectively, and (**c**) the used idealisations based on the material characterisation tests of the reinforcing bar (grey lines).

The impact of the material law employed to translate strains into stresses and further into bond shear stresses is discussed in the following, using half of the crack element between YS 3 and YS 5 at the two load steps marked in Figure 9c (*F* = 150 kN and 178 kN). Figure 10a,b top–bottom illustrate the steel strain, steel stress, and bond shear stress profiles at these load steps. Figure 10c shows three different idealisations of the stress–strain relationship of the reinforcement, which are used to explore the influence of the yield limit as defining parameter: in the green and red models, the yield limit was defined as the static and the dynamic value obtained in the material tests, respectively. In the blue model, the kink in the stress–strain relationship, as discussed in Section 4.3.2, was approximated by defining a line between the stress $\sigma_{s,k}$ where the Young’s modulus starts decreasing and the strain $\varepsilon_{sy}^{*}$ at the actual onset of yielding (Figure 10c). Finally, the bond shear stresses were calculated for the three resulting stress profiles using Equation (8). The results are plotted in the colours of the used material model (cf. Figure 10c). The strains were adjusted for the shrinkage as described in Section 5.1. The stresses were calculated using the idealisation for QST reinforcing bars as described by Equations (4)–(6).

As expected, the different models hardly have any influence on the results in the elastic range, except when the strain exceeds the strain $\varepsilon_{s,k}$, hereafter the flattening of the blue stress–strain relationship leads to minor stresses and stress gradients (Figure 10a, middle). This leads to a drop in the bond shear stresses (blue line Figure 10a, bottom). In contrast, a significant difference results for higher strains. In the load step under consideration, the strains of the crack element exceeded the yield limits ($\varepsilon_{sy,stat}$ and $\varepsilon_{sy}$) but were, except for areas close to the crack, below the hardening strain $\varepsilon_{sh}$. Using the green and red models, this leads to a plateau in the stresses (Figure 10b, middle) and therefore zero bond shear stresses (Figure 10b, bottom) over a major part of the crack element. The bond shear stresses only reappear next to the crack, where the strain surpassed the hardening limit $\varepsilon_{sh}$. The results of the third model (blue lines) show a stress variation over parts of the crack element and constant values around *x* = 65 to 105 mm, which results in zero bond shear stresses in this part as well. The vanishing bond shear stresses, contradicting the commonly postulated bond shear stress–slip relationships, are the result of the measured gradual strain increase from the yield limit to the onset of hardening, whereas steel stresses are presumed to remain constant in this range (yield plateau).

In a seminal paper, Shima et al. (1987) used strain gauges inside the reinforcing bar (as described in Section 1) to determine a bond stress–slip relationship in the elastic and plastic range [49]. In their experiments, they noticed a significant drop in the bond shear stresses when the yield strains were exceeded. A transition zone between the elastic and plastic regions of the bar, as reported here, could not be identified since they measured the strains at discrete points spaced at five bar diameters; the yielding location was thus not precisely known. They then fitted a strain distribution to the measured values, using a second-order polynomial equation and an immediate jump from the yield strain $\varepsilon_{sy}$ to the strain at the onset of hardening $\varepsilon_{sh}$, approximated the yield location at the centre between the last strain gauge with elastic strain and the first strain gauge with plastic values. The obtained strain distribution was then similar to the one illustrated in Figure 5a. This assumption allowed them to separate the bar into elastic and plastic regions such that an unambiguous inference of stresses from the strains was possible. However, the strain distribution obtained with DFOS suggests a different behaviour.

The disturbed regions with strains beyond the yield strain but inferior to the strain at the onset of hardening result in zero bond shear stress when applying the constitutive law presented in Section 4.3.2, or any other stress–strain relationship with a horizontal yield plateau. Modifying the stress–strain relationship measured for a bare bar by introducing a gradient in the yield plateau would cause bond shear stresses to appear in the plastic zone. However, their magnitude would depend on the assumed slope of the yield plateau, which is difficult to determine. Therefore, an unbiased determination of the local bond shear stresses close to the yield point currently appears impossible.

### 5.3. Pull-Out Test under Cyclic Loading

**Figure 11:**
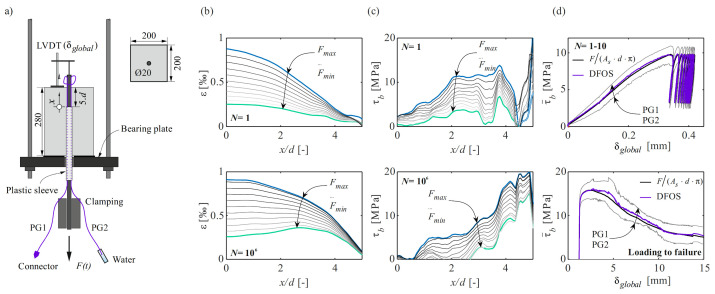
Pull-out test PO subjected to repeated tensile loading: (**a**) setup, geometry and instrumentation—dimensions in [mm]; (**b**) strain distribution along the bonded length for selected increasing load steps from $F_{min}$ to $F_{max}$ for first (top) and millionth (bottom) load cycle; (**c**) corresponding bond shear stress distribution and (**d**) comparison of the nominal bond shear stress and the mean value obtained from both fibre optical sensors PG1 and PG2 for the first ten load cycles (top) and the loading to failure after the millionth load cycle (bottom).

This section describes the results of a pull-out test (PO) according to the RILEM standard [56]. Figure 11a shows the test setup, geometry of the specimen and its instrumentation. The reinforcing bar was a straight QST bar of the class B500B according to the Swiss Standard SIA 262:2013, and a normal strength concrete ($f_{c}=28\;\mathrm{MPa}$) was used. The reinforcing bar was instrumented in a 180° configuration with polyimide-coated fibres (PG1 and PG2). In addition, the force *F* and the relative slip $\delta_{global}$ at the unloaded end were measured. A sinusoidal load was applied at the free end of the reinforcing bar, oscillating at a frequency of 2.5 Hz between 20 and 60% of the ultimate bond strength (defined as the average ultimate strength of three monotonic pull-out tests previously conducted). DFOS measurements were taken in intermediate cycles ran in displacement control. The gauge pitch was set to 1.3 mm and the resulting measurement rate was 40 Hz. The experimental procedure was similar to the one outlined in Section 3.1. Additional details on the experimental campaign can be found in [16].

Figure 11b,c illustrate the development of steel strains and bond shear stresses from the first (top row) to the millionth (bottom row) loading cycle. The former are obtained after removing outliers, replacing those by linearly interpolated values, and applying a moving average filter over 10.4 mm. Since loading was limited to the elastic range, the steel stress calculation was straightforward using the Young’s modulus determined in material characterisation tests. The bond shear stresses were calculated according to Section 4.3.3. It can be observed that between the first and millionth loading cycle, the bond shear stress profile redistributed over the bonded length: the peak of bond shear stresses increased and shifted toward the unloaded end of the reinforcing bar with an increasing number of load cycles.

To assess the plausibility of the results obtained with DFOS, the bond shear stress values were averaged over the bonded length and compared to the nominal values obtained by dividing the applied load by the perimeter of the bonded length of the reinforcing bar. Figure 11d shows the results for the first ten load cycles (top) and the loading up to failure (bottom) after the millionth cycle; note the different scales of the abscissa. The results show an excellent agreement between the two independent methods to determine the bond shear stresses.

With the use of DFOS, it could be shown that the bond shear stresses were far from being constant over the bonded length of a pull-out test as often presumed given the short embedment length. Furthermore, their profiles vary with increasing number of load cycles, redistributing toward the unloaded end. The quality of DFOS in this test was not affected by the repeated loading action.

### 5.4. Flexural Member

In Section 4.3 it was outlined how DFOS can be applied to determine the curvature, deflections and forces of an instrumented specimen. This procedure is demonstrated using a reinforcing bar of specimen Nn, a four-point bending test carried out in the realm of an experimental campaign studying lap splices with conventional and ultra-high performance fibre reinforced concrete [57]. Some results of this specimen, obtained from the instrumentation of the concrete compression zone, and specifications of the material properties are presented in the companion paper [28].

**Figure 12:**
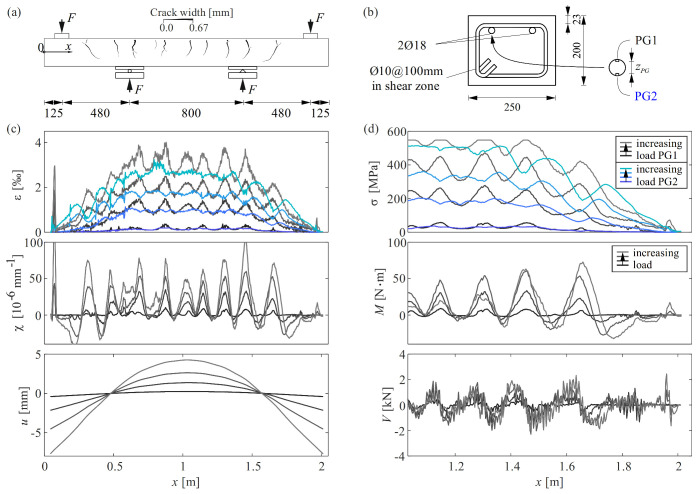
Fibre optical strain measurements on a flexural reinforcing bar of specimen Nn: (**a**) geometry and test setup with crack pattern and crack widths obtained with ACDM at the load *F* = 58 kN—dimensions in [mm]; (**b**) cross-section with instrumented reinforcing bar with two vertically aligned polyimide-coated fibre optical sensors glued inside a 1 × 1 mm^2^ groove (PG1 and PG2); (**c**) strains of both sensors and distribution of bar curvature and deflections derived from these strains over the entire length; and (**d**) calculated bar stresses from both FOS, and distribution of bending moment and shear force in the reinforcing bar over the right beam half.

Figure 12a,b illustrate the experimental setup, the geometry and reinforcement layout, and the instrumentation of the investigated reinforcing bar relevant in the present context, consisting of two vertically aligned polyimide-coated fibre optical sensors PG1 and PG2.

Figure 12c top–bottom shows (i) the strain profiles of both fibre optical sensors PG1 and PG2 for increasing load; (ii) the resulting curvature of the reinforcing bar

(9) $$\chi \left(x\right)=\left[\varepsilon_{s}^{PG1}\left(x\right)-\varepsilon_{s}^{PG2}\left(x\right)\right]/z_{PG}$$

obtained assuming plane sections remaining plane, where *z_PG_* *≈* *16* mm is the inner lever arm between sensors PG1 and PG2; and (iii) the reinforcing bar deflection

(10) $$u\left(x\right)=\int \int \chi \left(x\right)dx^{2}$$

(bottom) determined using the boundary conditions

(11) $$u\left(x=0.605\right)=0\;\mathrm{mm};\;u’\left(x=1.005\right)=0$$

Note that while the deflections of the reinforcing bar determined from Equation (10) corresponded well with the measured vertical displacements of the beam, determining the deflections of a beam from the curvatures of a reinforcing bar is not recommended in practice, as the goodness of the results is highly dependent on the accurate knowledge of the small distance between the sensors *z*_PG_.

Figure 12d top–down presents (i) the local stresses determined from PG1 and PG2 and the distribution of (ii) the bending moment of the bar, obtained again assuming plane sections remaining plane, and (iii) the shear force in the reinforcing bar.

The instrumentation proved to be suitable to determine all derived parameters and revealed that the curvature of the reinforcing bar was localised at the cracks, rather than being uniform over longer distances as commonly assumed. The bending moment and the shear forces along the bar also depend on the location of cracks: at cracked sections, the local bending (and curvature) of the bar is highest. In the shear zones, the curvature and hence, the bending moment change signs between cracks, and the shear forces exhibit discontinuities at the cracks, indicating that part of the applied shear force is carried by the reinforcing bar, which is known as dowel action. For instance, for the highest shown load (light grey curve), the shear force at the crack at *x* = 1.65 m changes from about 2.3 kN on the left side of the crack to −1.9 kN on its right side, corresponding to a shear force of 4.2 kN carried by the reinforcing bar at the crack; note that the global shear force at this loading stage amounted to 73 kN.

As expected, integral values, such as the bar deflection and the bending moment have a much smoother distribution than derivatives, such as the shear force in the reinforcing bar.

## 6. Conclusions

Distributed fibre optical sensing (DFOS) bears the potential to achieve a long-standing ambition of many researchers: to measure the internal strain distribution of RC structures with adequate resolution and effort, yet without biasing the behaviour. This paper, together with the companion paper [28], summarises the experience gained at the Chair of Concrete Structures and Bridge Design at ETH Zurich using DFOS in experimental research. Based on this, practical recommendations for instrumentation and data post-processing are provided, addressing both new and experienced users. Furthermore, the paper reports interesting findings achieved with DFOS and highlights potential challenges in deriving mechanically relevant quantities from the strain data.

For instrumenting reinforcing bars embedded in concrete, the use of fibres with a chemically bonded coating, placed inside a groove and covered with a two-component epoxy, has proven to lead to accurate results. However, the high spatial and temporal resolution makes it important to distinguish between global and local phenomena when analysing the acquired data and to select the size of the virtual sensor according to the target measurement. An example for measuring local deformations are the jagged strain distributions. As outlined in Section 3.2, in embedded reinforcing bars, these fluctuations are caused by the ribbed surface of the reinforcement as observed in bare bars [28], but superimposed by strain variations due to tension stiffening and amplified by the mechanical interlocking with the surrounding concrete. Applying a simple moving average filter with a filter size of at least one fold the rib spacing can mitigate these fluctuations. Optical fibres with a mechanically bonded coating deliver a smoother strain distribution but fail to measure sharp changes in the strain distributions.

The strain state of the reinforcement can be measured from the first contact with fresh concrete—provided that the fibre optical sensor is installed (i.e., a measurement of the initial backscatter is registered) before casting, such that subsequent measurements refer to the bare state. As discussed in Section 5.1, this renders it possible to account for the influence of the initial stress state caused by the hydration heat and shrinkage of the concrete on the reinforcement strains, which is particularly relevant in studies dealing with the deformation behaviour of structural concrete under service loads, such as tension stiffening, residual deformations after unloading, or the effect of repeated loading.

The strains induced in the fibres represent a combination of mechanically and thermally induced strains, as observed even in laboratory conditions (Section 3.1 and Section 5.1). In long-term tests and field monitoring, temperature compensation is therefore mandatory. When shielded from mechanical impact, the strains measured on completely loose fibre parts should display strain values approximating zero (no change compared to the reference state). Deviations from this value are mainly due to ambient temperature changes and can thus be used to compensate for the resulting strains. However, this implies the assumption that the host material experiences the same temperature change as the ambience. While this assumption is reasonable for thin specimens and small temperature gradients, a more sophisticated temperature compensation, such as the installation of unrestrained sensors inside the concrete, might be necessary in some cases.

In reinforced concrete elements, DFOS captures the variation of steel strains along an embedded reinforcing bar. The steel stresses can be obtained with knowledge of the stress–strain relationship, and the bond shear stresses follow from equilibrium. The slip, i.e., the relative displacement between concrete and reinforcing bar, is obtained by integrating the strains along the bar and neglecting or approximating the minor concrete displacements. A local bond stress–slip relationship can thus be obtained. However, the magnitude of the bond shear stresses remains sensitive to the post-processing methods applied and depends strongly on the material law used to convert the measured strains to stresses. While the derived values in the elastic and fully plastic range are reliable and could be shown to correspond on average to the nominal bond stress obtained in pull-out tests (Section 5.3), the conversion of strains close to the yield plateau of quenched and self-tempered reinforcing bars may lead to questionable steel and bond shear stresses (Section 5.2). This is because (i) the typically assumed constitutive law of this reinforcement might underestimate significantly the actual yield strain and (ii) when embedded in concrete the measured transition from elastic to plastic strains is not abrupt as is typically assumed [49], but occurs gradually according to the DFOS data. The reasons for the latter, in addition to its implications on structural behaviour, remain the subject of current research.

Repeated loading (Section 3.1 and Section 5.3) did not exhibit a detrimental effect on the performance of either fibre type investigated in this work.

## Figures and Tables

**Table 1:** Overview of the used fibre optical sensors in the presented experimental campaigns.

Coating Material	Model	Manufacturer/Reseller	Specimen	Fibre Denomination
Acrylate	Single-Mode Fibre E9/125	Huber & Suhner AG, CH	Steel plate, variable cross-section	AS
			Specimen d14c	AG
Polyimide	SM1500(9/125)P	Fibrecore, Southhampton, UK	Steel plate, variable cross-section	PG & PS
			Specimen d14c	PG
			RC tie ERH	PG1 & PG2
			RC tie ERV	PG1 & PG2
			Pull-out test PO	PG1 & PG2
			Beam Nn	PG1 & PG2

**Table 2:** Mechanical properties of the reinforcing steel bar used in specimens ERV and ERH, mean values and coefficient of variation.

Nominal bar diameter		[mm]	20
Effective diameter ^1^	$\emptyset_{eff}$	[mm]	19.95 (±0.1%)
Dynamic yield strength	$\sigma_{sy}$	[MPa]	558.5 (±1.0%)
Static yield strength	$\sigma_{sy,stat}$	[MPa]	536.2 (±1.1%)
Dynamic ultimate strength	$\sigma_{su}$	[MPa]	666.9 (±0.6%)
Static ultimate strength	$\sigma_{su,stat}$	[MPa]	626.1 (±0.7%)
Actual strain at onset of yielding	$\varepsilon_{sy}^{*}$	[‰]	3.78 ^2^
Strain at onset of hardening	$\varepsilon_{sh}$	[‰]	14.7 (±4.3%)
Young’s modulus	$E_{s}$	[GPa]	196.3 (±1.6%)

^1^ determined by weighing the reinforcing bars. ^2^ value determined on the investigated reinforcing bar.

## Data Availability

The data presented in this study are openly available in ETH Research Collection at https://doi.org/10.3929/ethz-b-000527485.
